# Severe Traumatic Brain Injury (TBI) Modulates the Kinetic Profile of the Inflammatory Response of Markers for Neuronal Damage

**DOI:** 10.3390/jcm9061667

**Published:** 2020-06-01

**Authors:** Cora Rebecca Schindler, Thomas Lustenberger, Mathias Woschek, Philipp Störmann, Dirk Henrich, Peter Radermacher, Ingo Marzi

**Affiliations:** 1 Department of Trauma, Hand and Reconstructive Surgery, University Hospital Frankfurt, 60596 Frankfurt, Germany; tom.lustenberg@gmail.com (T.L.); mathias.woschek@kgu.de (M.W.); philipp.stoermann@kgu.de (P.S.); d.henrich@trauma.uni-frankfurt.de (D.H.); marzi@trauma.uni-frankfurt.de (I.M.); 2 Institute of Anesthesiological Pathophysiology and Process Engineering, University Medical School, 89070 Ulm, Germany; peter.radermacher@uni-ulm.de

**Keywords:** multiple trauma, traumatic brain injury (TBI), risk prediction, biomarker, IL-6, IL-10, posttraumatic inflammation, S100b, NSE, GFAP

## Abstract

The inflammatory response plays an important role in the pathophysiology of multiple injuries. This study examines the effects of severe trauma and inflammatory response on markers of neuronal damage. A retrospective analysis of prospectively collected data in 445 trauma patients (Injury Severity Score (ISS) ≥ 16) is provided. Levels of neuronal biomarkers (calcium-binding Protein B (S100b), Enolase2 (NSE), glial fibrillary acidic protein (GFAP)) and Interleukins (IL-6, IL-10) in severely injured patients (with polytrauma (PT)) without traumatic brain injury (TBI) or with severe TBI (PT+TBI) and patients with isolated TBI (isTBI) were measured upon arrival until day 5. S100b, NSE, GFAP levels showed a time-dependent decrease in all cohorts. Their expression was higher after multiple injuries (*p* = 0.038) comparing isTBI. Positive correlation of marker level after concomitant TBI and isTBI (*p* = 0.001) was noted, while marker expression after PT appears to be independent. Highest levels of IL-6 and -10 were associated to PT und lowest to isTBI (*p* < 0.001). In all groups pro-inflammatory response (IL-6/-10 ratio) peaked on day 2 and at a lower level on day 4. Severe TBI modulates kinetic profile of inflammatory response by reducing interleukin expression following trauma. Potential markers for neuronal damage have a limited diagnostic value after severe trauma because undifferentiated increase.

## 1. Introduction

Polytrauma (PT) and severe traumatic brain injury (TBI) caused by road traffic accidents and falls are the main causes of death and disability in young patients under 45 years with immense socioeconomic impact through loss of productivity, medical and rehabilitation costs [1,2]. The treatment of patients with multiple organ injuries poses a particular challenge due to different injury patterns and severity, but also due to the complex immune response [3]. Post-traumatic exaggerated immunomodulation often leads to postinjury complications, multiple organ failure (MOF) or death and are predictors of mortality in trauma [4,5]. The brain is vulnerable to damage and failure due to its high metabolic rate and limited intrinsic energy reserve. The (neuro-)inflammatory environment subsequently leads to cell death and neurodegeneration in secondary brain damage but also to neuro reparative mechanisms in later stages. Secondary brain injury due to inflammatory processes is one of the main reasons for worsening of outcome [6,7,8]. Detecting the severity of injuries at an early stage, predicting their development and preventing secondary damage is of highest clinical interest, with biomarkers playing an important role [9]. One of the most studied serum biomarkers in TBI is the calcium binding protein B (S100B). The oligomeric cytoplasmic protein is predominantly found in astrocytes and involved in cellular processes and signal transduction [10]. Neuron-specific enolase 2 (NSE) is mainly found in the cytoplasm of neurons and neuroendocrine tissue and is involved in glycolysis in both neuronal cells and erythrocytes. Its detection in blood is considered as marker for neuronal damage [11,12]. The gliafibrillary acidic protein (GFAP) is a monomeric intermediate filament concentrated in the astroglial cytoskeleton. GFAP is brain-specific and is released into the peripheral blood circulation after death of astrocytes [13]. After multiple injuries and/or TBI, both the membrane integrity of brain cells and the integrity of the blood–brain barrier (BBB) is disturbed [14]. S100B, NSE and GFAP are released into the blood, with kinetic profile being related to injury pattern and (neuro-)inflammatory process [15,16]. Pro- and anti-inflammatory cytokines such as interleukin-6 and -10 (IL-6, IL-10) are released in response to tissue injury and lead to both reactive and restorative inflammatory processes [17]. Although interleukins are clear markers for immune activation, further investigation is needed to determine the extent to which they reflect brain damage as diagnostic factors [18]. In the acute phase reaction, IL-6 is known to regulate inflammation, immunity and neural development. An acute local and systemic release in brain tissue, blood and cerebrospinal fluid can be observed as a reaction to injury [19,20]. IL-10 is commonly known as an anti-inflammatory cytokine that performs immunomodulatory functions and is particularly important in the resorption phase. Its expression increases with the pathology of the Central Nervous System (CNS), promoting survival of nerve and glial cells and attenuating inflammatory responses [21]. Understanding the (neuro)immunological effect of TBI on the systemic inflammatory response is a key factor in the development of early targeted therapies [8]. Although several studies have investigated systemic cytokine levels in severely injured trauma patients [22,23] the specific influence of severe trauma or TBI on the neuronal and inflammatory response and expression of brain-specific biomarkers has not been described.

The aim of the present study was to characterize the systemic profiles of neuronal biomarkers S100b, NSE, GFAP and the pro- and anti-inflammatory markers IL-6 and Il-10 in severely injured patients and to assess the influence of severe TBI on the state of acute inflammation in these patients. Furthermore, it will be shown to what extent neuronal biomarkers used in TBI have sufficient significance to assess the influence of initial or indirect secondary intracranial damage in polytrauma.

## 2. Experimental Section

The study was performed at the University Hospital Frankfurt, Goethe University after approval by the Institutional Review Board (89/19) in accordance with the Declaration of Helsinki and following STROBE guidelines [24]. Written informed consent was obtained for enrolled patients or their legally authorized representatives in accordance with ethical standards.

We retrospectively reviewed a cohort of severely injured trauma patients admitted to the emergency department (ED) of the University Hospital of the Goethe University Frankfurt from 2012 to 2016. All clinical data were prospectively taken during the quality documentation of the TraumaRegister der Deutschen Gesellschaft für Unfallchirurgie (DGU)^®^, Berlin, Germany. All trauma patients in the ED were treated according to the Advanced Trauma Life Support (ATLS^®^ American College of Surgeons, Chicago, IL, USA) standard and the polytrauma guidelines [25]. Injury severity from trauma was calculated using the Injury Severity Score (ISS) based on the Abbreviated Injury Scale (AIS) score which assigns each injury a severity level between 1 (mild) and 6 (maximum) in different regions (head, face, thorax, abdomen, extremities and external injuries) [26,27]. The New Injury Severity Score (NISS) was calculated to better correlate the assessment measure with the polytrauma of the patients [28]. We evaluated 445 trauma patients with an ISS ≥ 16 between 18 and 80 years of age. Patients who met inclusion criteria were stratified into 3 groups: isolated severe TBI ((isTBI); AIS_head_ ≥ 4, all other body areas AIS ≤ 1, polytraumatized patients with severe TBI ((PT + TBI); AIS_head_ and AIS of other body area ≥3;) and polytraumatized patients without relevant brain injury ((PT); AIS_head_ ≤ 1;). Patients with known pre-existing immunological disorders, immunosuppressive medication, burns, concomitant acute myocardial infarction, thromboembolic events and patients who died within 5 days of hospital admission (incomplete serial blood samples) were excluded. Documentation containing further information on demography (age and sex) and injury.

Serial venous blood samples were obtained from traumatized patients on admittance to the ED through day 1–5 daily after trauma. Following baseline sample, subsequent blood was collected following the standard hospital procedures in pre-chilled ethylenediaminetetraacetic acid (EDTA) tubes (BD vacutainer, Becton Dickinson Diagnostics, Aalst, Belgium) and stored on ice. Blood was centrifuged at 2000× *g* for 15 min at 4 °C and the supernatant (serum) was stored at −80 °C until analysis. IL-6 and IL-10 concentrations were measured by IL-6/IL-10 Eli-pair Enzyme-linked Immunosorbent Assay ((ELISA); Diaclone, Hoelzel Diagnostica, Cologne, Germany) according to the manufacturer’s instructions. The following markers of brain injury were assessed using commercially available ELISA assays (Bio-Techne GmbH (R & D Systems GmbH, Wiesbaden, Germany): S100B (DY1820-05 Human S100B; assay range: 47–3000 pg/mL)), glial fibrillary acidic protein (DY2594-05 GFAP; assay range: 0.3–20 ng/mL) and neuron-specific enolase 2 (DY5169-05 Human Enolase 2/Neuron-specific Enolase; assay range: 78–5000 pg/mL), after sample dilution as needed.

Continuous normally distributed variables were summarized using means ± standard error of the mean (*SEM*), while categorical or continuous variables with skewed distributions were summarized using means ± standard deviation (*SD*). The *p*-values for categorical variables were derived from the two-sided Fisher’s exact test, and for continuous variables from the Mann–Whitney U test or the Kruskal–Wallis test. Significant values were adjusted by the Bonferroni post hoc test. Spearman’s rank correlation coefficients were calculated to determine correlations between inflammatory and neuronal biomarkers and injury characteristics. A *p*-value < 0.05 was considered to be statistically significant. Values are reported as means for continuous variables and as percentages for categorical variables. All analyses were performed using the Statistical Package for Social Sciences (SPSS for Mac^©^), version 26 (SPSS Inc., Chicago, IL, USA).

## 3. Results

### 3.1. Demographics and Clinical Injury Characteristics

Table 1 shows the demographic and clinical characteristics stratified by injury pattern. During the 5-year study period a total of 445 severely injured patients (ISS ≥ 16) was evaluated. A total of 104 patients met the inclusion criteria. Of these, 43 patients were severely injured without TBI (PT), 35 patients were polytrauma patients with severe TBI (PT+TBI) and 26 patients suffered from isolated severe TBI (isTBI). A total of 76.0% of the trauma patients were men. Patients with isolated TBI were significantly older (61 ± 3 years, *p* < 0.001) than those in other groups, about 70% of them were older than 55 years. Mean ISS of the patients with isolated TBI was significantly lower (24 ± 1, *p* < 0.01) but in NISS (*p* = 0.135) no significant difference was found. Computed tomographic findings of patients with PT severe TBI or isolated severe TBI usually suffered not only from one type of intracranial hemorrhage but from several entities simultaneously.

### 3.2. Systemic Profiles of Neuro Markers in Relation to Injury Pattern

**Figure 1:**
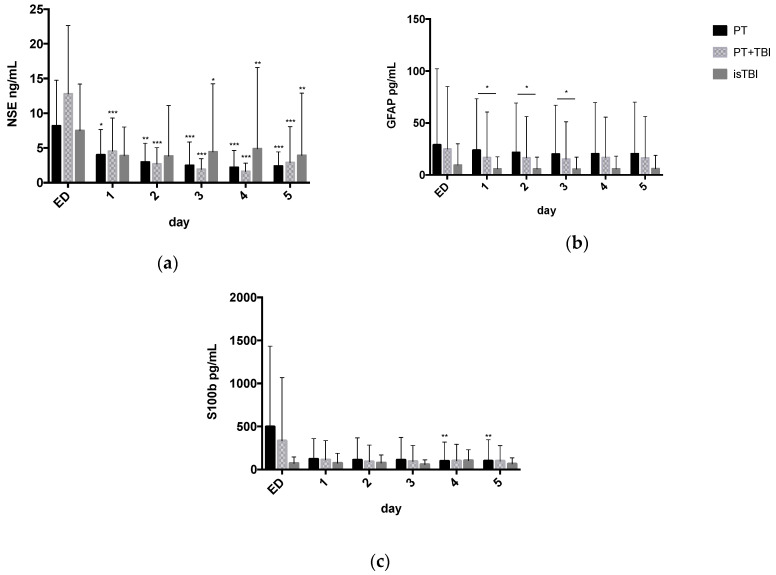
Neuronal serum marker levels (mean ± Standard Deviation) over the time course from admission to Emergency Department (ED) through to hospital day 5 stratified by injury pattern. * *p* < 0.05, ** *p* < 0.01, *** *p* < 0.001. (**a**) Neuron-specific enolase 2 (NSE) (**b**); glial fibrillary acidic protein (GFAP); (**c**) calcium-binding Protein B (S100b).

Figure 1 displays neuronal serum marker levels over the time course from ED admission to hospital day 5 stratified by injury pattern.

After trauma, the NSE expression is highest on the day of admission with maximum serum level of 8 ± 1 ng/mL (PT), 13 ± 2 ng/mL (PT + TBI) and 7 ± 1 ng/mL (isTBI) and steadily decreases over time.

Significantly higher GFAP expression was found in the PT cohort (day 1: 23.80 ± 7.94 pg/mL) comparing to isTBI (day 1: 5.82 ± 2.33 pg/mL, *p* = 0.038) 24 to 72 h after trauma. 

For S100b highest marker expression was found in PT patients (495 ± 150 pg/mL) on time of admission, followed by the PT + TBI group (333 ± 128 pg/mL). After isolated TBI the S100b expression was over all lower (71 ± 15 pg/mL) comparing PT and PT + TBI.

### 3.3. Systemic Profiles of Inflammatory Markers in Relation to Injury Pattern

**Figure 2:**
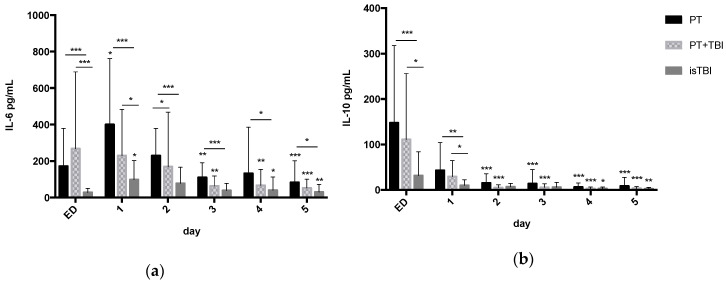
Inflammatory serum marker levels (mean ± Standard Deviation) over the time course from emergency department (ED) admission through to hospital day 5 stratified by injury pattern. * *p* < 0.05, ** *p* < 0.01, *** *p* < 0.001. (**a**) Interleukin (IL)-6 (**b**); Interleukin (IL)-10.

Figure 2 shows inflammatory serum marker levels over the time course from ED admission through to hospital day 5 stratified by injury pattern. 

In PT (day 1: 400 ± 71 pg/mL) and isTBI (day 1: 98 ± 22 pg/mL) group IL-6 peaked on hospital day 1 while in PT + TBI group highest IL-6 level was detected on admission (267 ± 86 pg/mL). All groups showed significant cytokine drop over time course. Over the entire time course, IL-6 levels were significantly higher in the PT cohort (*p* < 0.001) as well within the first 24 h in PT + TBI group (*p* < 0.001) compared to the isTBI group.

All groups showed highest IL-10 expression on admission (PT 148 ± 33 > PT + TBI 111 ± 30 > isTBI 31 ± 11 pg/mL) with significant decline from day 2 in the PT (− 71.2%) and PT + TBI (− 73.6%) groups and from day 4 in isTBI (− 9.5%) group. Within 24 h after trauma, IL-10-level was significantly higher in the PT (*p* < 0.001) and PT + TBI (*p* < 0.05) cohorts according to isolated TBI.

### 3.4. Relation between Marker Expression and Injury Severity

On admission we found significant positive correlation of increased IL-10 expression and injury severity (Spearman correlation (*rho*), ISS *r* = 0.4, *p* = 0.04; NISS *r* = 0.5, *p* = 0.01) as well as and positive correlation between increased cytokine expression (IL-10 *r* = 0.6, *p* = 0.001; IL-6 *r* = 0.5, *p* = 0.01) and higher risk of mortality (Revised Injury Severity Classification Score (RISC II)) in severely injured patients (PT) within 24 h after severe trauma (PT).

In PT + TBI group the S100b expression showed significant positive correlation with injury severity (ISS *r* = 0.4, *p* = 0.02), mortality risk (RISC II *r* = 0.4, *p* = 0.02) and severity of TBI (AIS_head_ *r* = 0.4, *p* = 0.05) up to 72 h after trauma.

In isTBI cohort we found negative correlation between GFAP expression and age (*r* = − 0.4, *p* = 0.03). After isolated TBI the inflammatory response correlated 24 h after trauma with the ISS (IL-6 *r* = 0.6, *p* = 0.01), NISS (IL-6 *r* = 0.6, *p* = 0.003; IL-10 *r* = 0.5, *p* = 0.04) and severity of head injury (AIS_head_/IL-10 *r* = 0.6, *p* = 0.002) as well as with risk of mortality (IL-10 *r* = 0.6, *p* = 0.004).

### 3.5. Inflammatory Status (IL-6/IL-10 Ratio) and Kinetic Profile of Neuro Markers

**Figure 3:**
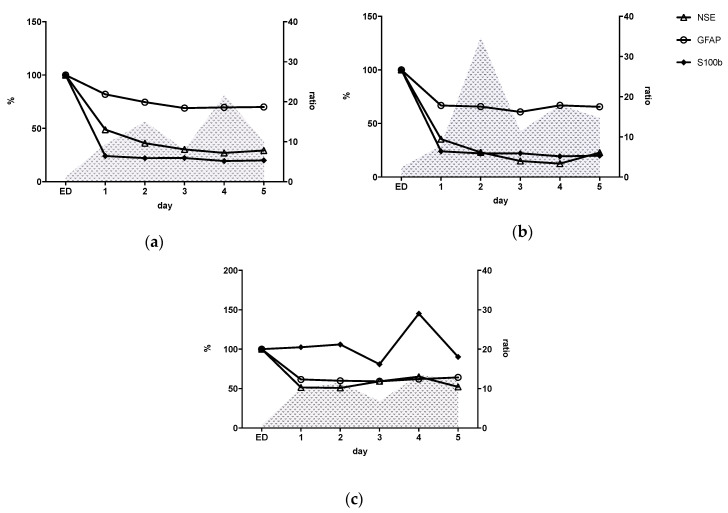
Pro-inflammatory response (Interleukin (IL)-6/Interleukin (IL)-10 ratio, grey-hatched) and neuronal marker expression (Neuron-specific enolase 2 (NSE), glial fibrillary acidic protein (GFAP), calcium-binding Protein B (S100b)) after (**a**) polytrauma (PT); (**b**) polytrauma + traumatic brain injury (TBI); (**c**) isolated TBI (isTBI). ED = Admission to Emergency Department.

Table 2 displays the relation between inflammatory response and neuronal markers expression stratified by injury pattern. Figure 3 shows inflammatory status (IL-6/IL-10 ratio) together with the kinetics of neuro marker expression during the time course. The comparison of the marker levels within a group in percentage ratio serves to illustrate the proportionality.

Kinetic profiles of PT and PT-TBI groups showed similar course from ED admission through to hospital day 5. While the PT and PT+TBI groups continued to decrease over time until 29.3% (PT) and 22.9% (PT + TBI), the TBI group showed constantly elevated NSE expression (52.3%) 5 days following trauma. All groups showed synchronous decline over time with a maximum reduction of GFAP expression to 64.2–70.0%. For S100b we found a drop down to 24.1% in PT and 33.9% in PT + TBI group within 24 h. After isolated TBI consistent S100b expression over time course (min. 80.9%, day 3) with a secondary peak on day 4 (145.0%) was found.

Based on the IL-6/IL-10 ratio we identified two phases of pro-inflammation for all groups: increase from admission to day 3 with peak after 48 h and secondary from day 3 to day 5 with peak on day 4. The pro-inflammatory quotient after 48 h was higher in the PT + TBI group (ratio = 34.8) than after PT (ratio = 15.0) and isTBI (ratio = 11.6). The lowest pro-inflammatory reaction was found for isTBI.

### 3.6. Relation between Inflammatory Response and Neuronal Markers

After severe PT a significant correlation was found between IL-6 and IL-10 level (*r* = 0.8, *p* < 0.001) on admission and day 1 to day 5. Over the entire time course, a positive correlation was observed between S100b and NSE and IL-6 expression with statistical significance from day 4 (NSE *r* = 0.04, *p* = 0.01, IL-6 *r* = 0.4, *p* = 0.04) and GFAP.

After PT with severe TBI, GFAP and S100b expression showed statistically significant synchronous decrease (*r* = 0.3, *p* = 0.04) over the entire time course. Positive correlation was found as well between NSE and S100b (*r* = 0.3, *p* = 0.04) from day 3.

A positive relation of GFAP with NSE (admission: *r* = 0.3, *p* = 0.05) and with S100b (*r* = 0.5, *p* = 0.001) levels over the entire time course and between NSE (*r* = 0.4, *p* = 0.01) and with S100b on admission, day 2, 3 and 5 was measured in patients with isTBI.

## 4. Discussion

Of 104 patients included, 75% were male and the cohort with isolated TBI was significantly older than the PT and PT+TBI group. Polytrauma is the leading cause of death in Western countries for people up to 45 years of age, in a male-to-female ratio of 2.6:1. Older patients cause most TBI-related hospitalizations and deaths [1,29]. In this study it was shown that the application of both the already used and the more recent biomarkers S100b, NSE and GFAP have limited value in the assessment of TBI in polytraumatized patients. Nevertheless, biomarkers are certainly the optimal complement to clinical and radiological findings to assess the course and outcome of the injury sufficiently. Biomarkers can reveal the extent of cell death, provide early indications of occult or later visible organ damage, and thus have an immense clinical value.

### 4.1. S100b Missing Diagnostic Value after Severe Trauma

One of the most often studied serum biomarkers in TBI is S100b [7]. Due to its high molecular weight, the protein is only able to pass through the BBB, if it has an increased permeability caused by injury [14]. Therefore, this marker was used to detect (sensitivity 81–100%) and predict the outcome of a severe TBI. The potential pitfalls of S100b in the different areas are usually related to its specificity (20–67%) and sensitivity in the detection and assessment of intracranial injury. Consistent cut-off levels (51 pg/mL to 210 ng/mL at admission) are difficult to find in the literature [10,12,15]. In this study, the highest serum levels for S100b were measured in polytrauma patients (495 ± 150 pg/mL) at admission, followed by the PT + TBI group (333 ± 128 pg/mL) and isolated TBI (71 ± 15 pg/mL). S100b is thus expressed lowest after isTBI, although not significantly. S100b is also expressed in peripheral nervous tissue in addition to the CNS. Furthermore, muscle tissue, chondrocytes and adipocytes were identified to release S100b after injury [3]. TBI could only be detected with a predictive power of 67% in pediatric polytrauma patients with TBI and fracture of long bones [30]. Orth-Nissen et al. found higher serum levels of S100b in patients with multiple injuries than in patients with isolated TBI or without TBI. This suggests that elevated serum concentration of S100b after trauma seems to be significantly influenced by an extra-cerebral injury and is an indicator for a global tissue and organ damage [31]. It was shown that pro-inflammatory cytokines such as IL-6 induce an increase of S100b and GFAP secretion via the mitogen-activated protein kinase (MAPK) pathway [32]. Furthermore, a positive correlation between neuro marker expression over time was found after PT + TBI and isolated TBI. Possibly this is an indicator of congruent marker expression after neuronal damage in TBI.

### 4.2. Non-Specific Increase of NSE Expression by Cofounding Effect after Severe Trauma

NSE is localized in the cytoplasm of neurons, erythrocytes, platelets and neuroendocrine cells. It has been discussed as a biomarker for neuronal damage and has been associated with the severity of injury and clinical outcome after severe TBI. In the literature, serum levels after trauma ranged between 6.5–21.2 ng/mL [9,12,30]. In this study it was shown that after polytrauma with TBI, the NSE expression is highest on the day of admission with a maximum serum level of 13 ± 2 ng/mL (PT + TBI) > 8 ± 1 ng/mL (PT) > 7 ± 1 ng/mL (isTBI) and decreases steadily until day 5. A possible explanation for this finding could be an NSE release due to hemolysis after polytrauma. Another possibility might be the release of neuronal NSE due to reperfusion damage in the course of polytrauma with subsequent BBB disruption by immune reaction. Serum protein S100b and NSE increased temporarily as a result of multiple trauma associated with hemorrhagic shock might lead to cerebral hypoperfusion and brain damage in porcine model [33,34]. Due to its expression in neuroendocrine cells NSE is also studied in association with malignant and inflammatory lung diseases and first studies analyzing NSE release after lung injury have been published [35,36]. Methodological and technical problems such as slow elimination and potential artifacts in trauma patients call into question their diagnostic value as a screening tool [33]. It is striking that the isTBI group still shows an increased NSE expression (isTBI 52.3% > PT + TBI 22.9%, PT 29.3%) 5 days after the trauma, which may be an expression of severe neuronal damage during the secondary brain swelling and injury.

### 4.3. GFAP Expression Is Induced by Multiple Injuries

GFAP was largely considered brain-specific and is released into the peripheral bloodstream after death of astrocytes [11]. Meanwhile GFAP expression is also described in Schwann cells, myoepithelial cells, chondrocytes and fibroblasts [37]. An acute intracerebral hemorrhage leads to an immediate mechanical destruction of the astroglia with subsequent release of GFAP into the bloodstream [38]. According to Lei et al., human GFAP serum levels are elevated from admission and during the first 5 days after severe TBI and are predictive of the neurological outcome after 6 months. In the literature, different GFAP cut off levels from 6.8 pg/mL to 0.7 ng/mL are described after TBI for unfavorable outcome [13,39]. In the present study, significantly higher GFAP levels in the PT group (day 1: 24 ± 8 pg/mL) compared to isTBI (day 1: 6 ± 2 pg/mL, *p* < 0.05) were measured 24 to 72 h after trauma. Hsieh et al. demonstrated that over a 6 h period of reperfusion after intestinal ischemia, microglial cells and astrocytes were significantly activated in the brain [40]. Polytrauma increases GFAP and neutrophil expression and leads to secondary brain damage in the presence of exacerbated neuroinflammation, edema and disruption of the BBB [41]. In the isTBI cohort, a negative correlation between GFAP expression and age (*r* = − 0.4, *p* = 0.03) was found. It is possible that GFAP is less expressed by the significantly older isTBI patients.

### 4.4. Severe Traumatic Brain Injury (TBI) Modulates Kinetic Profile of Inflammatory Response

The acute inflammatory reaction plays an important role in immune defense, but can also exert serious adverse consequences, if the excessive immune response leads to systemic inflammation, secondary organ damage and subsequent MOF [3,21]. After TBI, activation of glial cells, microglia and astrocytes as well as infiltration of blood leukocytes takes place within minutes, releasing pro- and anti-inflammatory mediators and various growth factors and triggering a local neuroinflammation as well as a systemic inflammatory response. In this case, the brain functions as both target and effector organ [42]. Among these mediators, interleukins play a particularly prominent role in the development of posttraumatic complications [18,21]. IL-6 is secreted in increased amounts early after trauma. Serum concentrations in available studies range from 47 to 245 pg/mL [8,19,43]. Following TBI, IL-6 levels increase with peak concentrations of 93 to 269 pg/mL in human serum and IL-6 levels greater than 100 pg/mL in the first 24 h after trauma have been associated with severe brain injury [8,18,44]. In this study, IL-6 expression in polytraumatized patients with and without concomitant head injury was significantly higher compared to isTBI (day 1: PT 400 ± 71, PT + TBI 230 ± 53 pg/mL, > isTBI 98 ± 22 pg/mL, *p* < 0.001); all groups showed a relevant cytokine decrease (*p* < 0.001) over time.

IL-10 is an anti-inflammatory cytokine that regulates and limits acute inflammation in response to trauma to prevent tissue damage, such as secondary brain damage after TBI [21,45]. IL-10 plays a crucial role in inflammatory and autoimmune diseases of the intestine, chronic infections, tumor development, neuroinflammation, -degeneration as well as multiple organ dysfunction syndrome (MODS) and MOF after polytrauma, where IL-10 levels from 21.0 to 340.7 pg/mL were shown [42,46]. In this study, the highest IL-10 levels were measured on admission and a continuous decrease until day 5 was shown. The overall increase within the first 24 h was significantly higher in PT (148 ± 33 pg/mL, *p* < 0.001) and PT+TBI (111 ± 30 pg/mL, *p* < 0.05) than after isTBI (31 ± 11 pg/mL). Previous studies described a correlation of IL-6 with the severity of injury (ISS) or insult and mortality, especially during the first post-traumatic hours. IL-6 is associated with the occurrence of MODS and sepsis together with IL-10 within 24 h after trauma [23]. In the present study, IL-10 was positively correlated with ISS and NISS on the day of admission and day 1.

In addition, a positive correlation between IL-6 and IL-10 expression with the mortality risk (RISC II) was shown. Thus, the inflammatory reactions are most likely associated with the severity of injury (ISS: PT + TBI 37 > PT 30 > isTBI 24, *p* < 0.01) and the extent of tissue trauma itself. Interestingly, polytraumatized patients with severe head trauma showed—although not statistically significant—lower IL-6 values than severely injured patients without TBI despite a higher ISS. This corresponds to already published results from 2016, which raised the question of an immunosuppressive role of TBI [8]. With increasing severity of TBI (AIS_head_ isTBI 4.5 > + TBI 4.1 > PT 0.3), an overall lower cytokine expression was observed. Based on the IL-6/IL-10 ratio, two phases of pro-inflammation can be identified for all three groups from hospital admission to day 3 with a first peak after 48 h and from day 3 to day 5 with a second peak on day 4. Overall, the pro-inflammatory quotient was higher after PT and PT + TBI than after isTBI, with the first phase being strongest after PT + TBI. It is possible that the limited cytokine expression and depressed pro-inflammatory ratio indicates an immunomodulatory effect after severe TBI, but the pro-inflammatory immune response was ultimately relatively strongest after PT + TBI. Data on the predictive ability of IL-6 serum and the neurological outcome are limited and contradictory. While in the pediatric TBI serum IL-6 showed no association with the neurological outcome [47], others showed that high IL-6 correlates with a poor neurological outcome [44]. The determination of cytokine levels can serve not only as a prognostic value for the development of complications, but also for the early identification of patients who are at risk of suffering a “second hit” by renewed immune stimulus in the form of a secondary intervention.

An important pathophysiological factor in the development of posttraumatic complications is the dysfunction of the external (skin) and internal paracellular blood and organ barriers, including the brain (BBB), air and gut blood barriers, resulting in tissue flooding with immune cells and microbial invasion [48]. IL-6 modulates the expression of tight junction proteins in cerebral microvasculature of sheep, and the release of adhesion molecules in plasma of polytrauma patients (ISS ≥ 18) correlates with disease severity and organ dysfunction [49]. Furthermore, the hemorrhagic shock is associated with barrier dysfunction of the gut and development of MODS after the trauma and can lead to a traumatic endotheliopathy by glycocalyx degradation, like in the CNS [50]. Studies have shown that paracellular hyperpermeability due to inflammatory reactions and abnormal release of neurotransmitters is the basis for intestinal barrier dysfunction after TBI [51]. The increased permeability of the organ barriers allows metabolites to enter the bloodstream and to detect specific biomarkers [14]. Identifying the severity of injuries at an early stage, predicting their development in order to prevent secondary damage is of paramount interest in trauma treatment, with the detection of biomarkers playing an important role [43].

### 4.5. Limitations of the Study

The most important limitation is the retrospective nature of the data analysis; however, all clinical data were acquired in a prospective manner, and only the cytokines and the neuro-specific markers were measured later-on. Furthermore, patients with isolated TBI were significantly older than the comparison groups. In a recently published multicenter study, older patients with blunt trauma showed significantly lower plasma cytokine and chemokine concentrations, including IL-6 levels, than patients <55 years of age [52]. The patients included in the isTBI cohort also suffered from several types of intracranial bleeding simultaneously, which does not allow differentiation between bleeding entities and marker expression. In the present study we found a difference between patient’s age of the PT and PT+TBI vs. isTBI group and in their interleukin expression. Ultimately, no conclusions can be drawn as to how age may have altered the inflammatory response. The variance of the individual values is large, specifically of neuronal markers, which is due to potential confounders such as volume administration, blood products, shock, which are difficult to standardize.

## 5. Conclusions

Although no significant correlation between cytokine expression and release of neuronal markers after trauma could be shown, it is clear that the application of the biomarkers NSE, GFAP and S100b have limited value in the assessment of TBI in polytrauma patients, the levels are not substantially elevated. Possible cause could be a modulation of the kinetic profile of the inflammatory response following severe traumatic brain injury (TBI). IL-6 and IL-10 levels were lower in patients with multiple injuries and concomitant TBI compared to those without severe TBI, suggesting a reduced visible systemic inflammatory response due to traumatic brain injury. In addition, significant correlations were shown between IL-6 and IL-10 levels and injury severity after TBI, indicating the general posttraumatic inflammatory course.

## Figures and Tables

**Table 1:** Demographic and clinical injury characteristics stratified by injury pattern.

	PT (*n* = 43)	PT + TBI (*n* = 35)	isTBI (*n* = 26)	*p*-Value
**Male**	69.8%	80.0%	80.8%	
**Age (y, ± SEM)**	46 (±2)	43 (±3)	61 (±3)	<0.001
**Age ≥ 55 years**	32.6%	25.7%	69.2%	<0.001
**ISS (±SEM)**	30 (±2)	37 (±2)	24 (±1)	<0.01
**ISS ≥ 25**	74.4%	77.1%	53.9%	<0.01
**NISS (±SEM)**	37 (±2)	44 (±2)	38 (±2)	0.135
**AIS_head_ (±SEM)**	0.3 (±0.1)	4.1 (±0.2)	4.5 (±0.1)	<0.001
**AIS_chest_ ≥ 3**	81.4%	68.6%	0.0%	<0.001
**AIS_abdomen_ ≥ 3**	46.5%	11.1%	0.0%	<0.001
**AIS_extremity_ ≥ 3**	51.2%	37.1%	0.0%	<0.001
**ICH (%)**		57.1%	61.5%	0.730
**SDH (%)**		37.1%	61.5%	0.059
**SAH (%)**		34.3%	57.7%	0.069
**EDH (%)**		20.0%	11.5%	0.377

Abbreviations: PT = Polytrauma, isTBI = isolated Traumatic Brain Injury, SEM = Standard Error of Mean, y = years, ISS = Injury Severity Score, NISS = New Injury Severity Score, AIS = Abbreviated Injury Score, ICH = intracerebral hemorrhage, SDH = subdural hematoma, SAH = subarachnoid hemorrhage, EDH = epidural hematoma.

**Table 2:** Relation between inflammatory response and neuronal marker expression.

		PT					PT + TBI					isTBI				
Day		GFAP	S100ß	IL6	IL10	Ratio	GFAP	S100ß	IL6	IL10	Ratio	GFAP	S100ß	IL6	IL10	Ratio
ED	NSE	0.21	0.06	0.024	0.00	0.12	0.13	−0.05	0.37	0.46 *	− 0.12	0.55 **	0.44 *	−0.17	−0.00	−0.30
	GFAP		0.08	0.18	0.06	0.04		0.30	−0.11	−0.14	−0.06		0.27	−0.03	0.25	−0.58 *
	S100ß			−0.06	0.01	−0.08			−0.14	0.06	−0.31			0.05	0.09	−0.23
	IL6				0.78 ***	0.39				0.64 **	0.31				0.76 ***	−0.19
	IL10					−0.21					−0.50 *					−0.80 ***
1	NSE	−0.14	0.32 *	0.09	0.29	−0.15	0.17	0.07	0.12	−0.35	−0.29	0.44 *	0.12	0.23	0.39	−0.30
	GFAP		0.17	0.18	−0.08	−0.09		0.52 **	0.08	0.27	−0.28		0.54 *	−0.03	0.03	−0.31
	S100ß			−0.12	−0.7	−0.06			0.13	0.27	−0.28			0.18	−0.10	0.00
	IL6				0.41 *	0.34				0.48 *	0.33				0.66 **	0.28
	IL10					−0.64 ***					0.61 **					−0.66 **
2	NSE	−0.16	0.23	0.33	0.00	0.07	0.29	0.23	0.06	0.17	0.11	0.63 *	0.22	0.18	0.08	0.06
	GFAP		0.30	−0.12	0.38	−0.53 *		0.46 *	0.23	0.20	−0.20		0.48 *	−0.05	−0.14	−0.07
	S100ß			−0.25	0.10	0.15			0.06	0.27	0.42			0.25	0.24	−0.18
	IL6				0.08	0.31				0.28	0.61 *				0.33	0.56 *
	IL10					−0.85 ***					−0.22					−0.69 **
3	NSE	0.02	0.21	0.20	−0.08	−0.02	0.24	0.23	0.15	0.18	0.04	0.54 *	0.48 *	0.03	−0.05	−0.17
	GFAP		0.26	−0.20	0.24	0.17		0.43 *	0.36	0.27	−0.10		0.50 *	−0.24	−0.17	−0.25
	S100ß			−0.05	−0.15	0.44			0.03	0.42 *	−0.28			−0.05	−0.87	−0.08
	IL6				0.49 *	0.26				0.74	0.46				0.34	0.38
	IL10					−0.7 ***					−0.78 ***					−0.44
4	NSE	−0.02	0.27	0.07	0.01	−0.08	0.24	0.17	0.15	−0.74	−0.00	0.55 *	−0.10	−0.04	−0.06	0.48
	GFAP		0.26	−0.06	0.22	−0.32		0.44 *	0.36	0.16	0.21		0.17	−0.10	−0.01	0.25
	S100ß			0.4 *	0.03	0.22			0.10	0.28	0.17			0.08	0.03	0.06
	IL6				0.38	0.76 ***				0.01	0.75 **				0.53 *	0.84 **
	IL10					0.21					−0.23					0.04
5	NSE	0.00	0.31	0.07	0.12	−0.20	0.03	0.25	−0.03	−0.02	0.10	0.56 *	0.22	−0.07	−0.16	0.73 **
	GFAP		0.27	−0.20	0.00	−0.02		0.49 *	0.04	0.27	−0.36		0.30	−0.17	−0.30	0.54
	S100ß			0.42 *	0.31	0.21			−0.11	0.42	−0.35			0.21	−0.22	0.31
	IL6				0.65 ***	0.52 *				0.42	0.72 **				0.68 ***	0.21
	IL10					0.34					−0.71 **					0.24

Spearman correlation (*rho*), * *p* < 0.05, ** *p* < 0.01, *** *p* < 0.001. Abbreviations: PT = Polytrauma, isTBI = isolated Traumatic Brain Injury, ED = Admission to Emergency Department, NSE = Neuron-specific enolase 2, GFAP = glial fibrillary acidic protein, S100b = calcium-binding Protein B.
